# Phase II trial of imiquimod and HPV therapeutic vaccination in patients with vulval intraepithelial neoplasia

**DOI:** 10.1038/sj.bjc.6605611

**Published:** 2010-03-16

**Authors:** S Daayana, E Elkord, U Winters, M Pawlita, R Roden, P L Stern, H C Kitchener

**Affiliations:** 1 Academic Unit of Obstetrics and Gynaecology, University of Manchester, St Mary's Hospital, Whitworth Park, Manchester M13 0JH, UK; 2 Cancer Research UK Immunology Group, Paterson Institute for Cancer Research, University of Manchester, Christie Hospital NHS Trust, Manchester M20 4BX, UK; 3 Department of Genome Modifications and Carcinogenesis, German Cancer Research Centre (DKFZ), Im NeuenheimerFeld 280, Heidelberg, Germany; 4 Department of Pathology, Johns Hopkins University, Baltimore, MD 21231, USA

**Keywords:** vulval intraepithelial neoplasia (VIN), imiquimod, therapeutic HPV vaccination, T regulatory cells

## Abstract

**Background:**

Vulval intraepithelial neoplasia (VIN) is a premalignant condition, which is frequently associated with type HPV16 infection, and multifocal disease has high rates of surgical treatment failure.

**Methods:**

We report a phase II clinical trial of the topical immunomodulator, imiquimod, for 8 weeks, followed by 3 doses (weeks 10, 14 and 18) of therapeutic human papillomavirus (HPV) vaccination (TA-CIN, fusion protein HPV16 E6E7L2) in 19 women with VIN grades 2 and 3. Histology and HPV testing of biopsies were performed at weeks 0, 10, 20 and 52. Intralesional infiltration of T-cell subsets and lymphocyte proliferation for HPV systemic immune responses were also assessed.

**Results:**

Lesion response (complete regression of VIN on histology) was observed in 32% (6 out of 19) of women at week 10, increasing to 58% (11 out of 19) at week 20 and 63% (12 out of 19) at week 52. At this time, 36% (5 out of 14) of lesions showed HPV16 clearance and 79% (15 out of 19) of women were symptom free. At week 20, after treatment with imiquimod and vaccination, there was significantly increased local infiltration of CD8 and CD4 T cells in lesion responders; in contrast, non-responders (persistent VIN by histology) showed an increased density of T regulatory cells. After vaccination, only lesion responders had significantly increased lympho-proliferation to the HPV vaccine antigens.

**Conclusion:**

The therapeutic effect of treatment depends on the differential immune response of responders and non-responders with affect locally and systemically.

Vulval intraepithelial neoplasia (VIN) is increasing in incidence, and more so in younger women. A >400% increase in the incidence of VIN has been reported between 1973 and 2000, whereas the corresponding increase in incidence of vulval cancer has only been 20% (Akerman *et al*, 2007). This probably reflects an increasing incidence of human papillomavirus (HPV) high-risk type genital tract infections, as the most common type of VIN (usual or undifferentiated) shows over 85% HPV positivity, with type 16 DNA being detected in 75% of high-grade VIN (De Vuyst *et al*, 2009). HPV infection of the lower genital tract and its outcome are influenced by host immune response, virulence of the HPV, smoking and immunosuppression (Duong and Flowers, 2007; Sherman *et al*, 2008). Successful adaptive immune responses that lead to clearance of genital HPV infection are believed to be mediated by local T cell-mediated immunity (Farhat *et al*, 2009). The virus life cycle and several immune evasion mechanisms limit virus-specific immunity, facilitating persistent infection and the increased risk of carcinogenesis (Stern, 2005). Indeed, it has been shown that patients with persistent HPV infection have depleted T-cell responses against the viral early gene products E2 and E6 measured systemically (de Jong *et al*, 2004; Farhat *et al*, 2009). In addition, T regulatory cells are negative regulators of otherwise useful anti-tumour T-cell immunity and locally increased levels have been implicated in the persistence of both cervical and vulval HPV-associated lesions (van der Burg *et al*, 2007; Winters *et al*, 2008).

VIN, which is often multifocal and extensive, has been traditionally treated by surgical excision or laser ablation, but such therapies are associated with high rates of recurrence (Jones *et al*, 2005). Unfortunately, for patients with chronic VIN, younger age and extent of surgery correlate directly with psychological morbidity and poorer sexual function (Thuesen *et al*, 1992; Likes *et al*, 2007; Shylasree *et al*, 2008). The natural history of VIN can be viewed as a struggle between HPV-driven premalignant intraepithelial neoplasia and immune control mechanisms. With time, the selection of cells with a malignant phenotype and ability to escape the immune control can result in vulval cancer. Non-surgical management of VIN with anti-viral and anti-neoplastic agents has had limited success (Vilmer *et al*, 1998; Tristram and Fiander, 2005), but a rational therapeutic strategy for chronic VIN could aim to alter the local immune response in favour of clearance of persistently HPV-infected cells.

Several different clinical studies influencing local and/or systemic immunity to HPV using imiquimod, photodynamic therapy (PDT) and therapeutic HPV vaccination in the management of VIN have been reported. Imiquimod, an immune response modifier that exerts an effect through a Toll-like receptor (TLR), (Schon and Schon, 2007) can stimulate not only local innate immunity (non-specific) with potent anti-tumoural effects but also drive an adaptive immune response (e.g., specific T-cell effectors) in secondary lymphoid tissues by activating tissue antigen-presenting cells. In a recent randomised controlled trial, treatment of VIN with imiquimod twice weekly for 16 weeks resulted in a ⩾25% lesion size reduction at 20 weeks in 81% of patients compared with none in the placebo group (van Seters *et al*, 2008). PDT uses a combination of light and photosensitiser drug to damage tumour tissue by modifying cellular functions, inducing cell death by necrosis or apoptosis, and encourages inflammation and anti-tumour immunity (Castano *et al*, 2006). Studies using PDT have shown good symptom and clinical response in patients with VIN (Martin-Hirsch *et al*, 1998; Fehr *et al*, 2001; Olejek *et al*, 2008). A recent trial of combined treatment with imiquimod followed by PDT (Winters *et al*, 2008) showed an overall lesion response rate of 55%, with 30% showing complete lesion resolution at 52 weeks. In this study, non-responders showed a significantly higher level of T regulatory cells in the lesions after imiquimod treatment (Winters *et al*, 2008). Such response rates are clinically relevant, and the treatment regimen was feasible for the majority. Non-responders to imiquimod seem to be relatively refractory, and this may derive from their unfavourable local immune environment, in particular, increased proportions of T regulatory cells, limiting the action and development of any HPV T-cell immunity. Therapeutic HPV vaccines are designed to generate cell-mediated immunity against HPV-infected cells that express the early viral proteins E6 and E7, which exert an effect as oncogenes and also tumour-specific antigens. Clinical trials using either PDT or therapeutic HPV vaccination have shown an association between clinical responses and tumour-infiltrating lymphocytes (TILs) (Abdel-Hady *et al*, 2001; Davidson *et al*, 2003a).

Optimal therapeutic immunity results from the interaction of nonspecific innate immunity and antigen-specific adaptive immunity. Stimulation of the local innate immunity can have a direct controlling effect on virally infected cells as well as attracting local infiltration of TILs (Dermime *et al*, 2002). The challenge is to get the most useful balance of helper (CD4) and cytotoxic (CD8) T-cell infiltration, which are associated with positive prognosis, and minimise the T regulatory cell infiltration, which is known to be associated with poor outcome, as shown in several studies of gynaecological cancer (Kondratiev *et al*, 2004; Sato *et al*, 2005; Wolf *et al*, 2005; van der Burg *et al*, 2007). The rationale of this study was that an initial local imiquimod treatment, in addition to having a direct effect on VIN, can also provide an immunological platform for the therapeutic HPV vaccination to achieve an enhanced and durable response.

In this phase II trial, we used a combination of imiquimod and vaccination with TA-CIN, which is a subunit vaccine comprising a HPV16 E6E7L2 fusion protein, proven safe and immunogenic in previous phase I and II trials (de Jong *et al*, 2002; Davidson *et al*, 2004; Smyth *et al*, 2004).

## Patients and methods

Women aged 18–70 years with biopsy-proven VIN grades 2 and 3 were recruited between March 2006 and May 2007. Exclusion criteria were pregnancy, invasive disease, immunosuppression, history of severe allergy and previous HPV vaccination. Imiquimod 5% cream was self-administered for 8 weeks, escalating from one application in week 1 to two in week 2 and three applications in weeks 3–8. This was followed by three intramuscular doses of TA-CIN (1 ml of 128 *μ*g ml^–1^) at weeks 10, 14 and 18. The primary objective was to measure treatment effect on VIN by lesion size and histology and the secondary objectives were to assess lesion HPV status, symptoms, immune responses as well as safety, toxicity and tolerability. Women were reviewed at weeks 0, 10, 14, 18, 20, 26 and 52, the primary end point. Punch biopsies were taken for histology and HPV typing at weeks 0, 10, 20 and 52. Heparinised blood (40 ml) was obtained for immunological assays at weeks 0, 10 and 20.

### Clinical response

Complete regression of VIN on histopathology of an index lesion(s) was designated the lesion response. Histological assessment was performed on formalin-fixed punch biopsies. Biopsies were analysed for HPV typing by reverse line blot assay, which is able to amplify and detect 37 HPV genotypes (Gravitt *et al*, 1998). Safety, tolerability and toxicity were assessed clinically, supplemented by FBC and serum biochemistry at weeks 0, 10 and 20. A full record of adverse events was kept. Patients were advised to maintain a symptom diary in a visual analogue scale form. This was reviewed at clinic visits when patient assessment of vulval pain, itch, swelling, discharge and/or any other symptoms were graded as none, mild, moderate or severe. Regression of symptoms from moderate or severe (interfering with lifestyle) to mild or none (not interfering with lifestyle) categories was considered to be a symptom response.

### Immunofluorescence

Local immune responses were quantified by assessing the TIL density on 7 *μ*m sections of the punch biopsy frozen in liquid nitrogen and stored at −80 °C. TILs from five representative fields of image were counted for each section. T regulatory cells were identified by double immunofluorescence labelling for FoxP3 and CD4. This was performed with mouse IgG2a anti-CD4 (Abcam, Cambridge, UK) at a concentration of 1 : 100 and mouse IgG1 anti-FOXP3 (eBioscience, Hatfield, UK) at a concentration of 1 : 50 and respectively detected using goat antimouse IgG2a Alexa Fluor 546 3 *μ*l per 1000 *μ*l and goat antimouse IgG1 Alexa Fluor 488 at 3 *μ*l per 1000 *μ*l (Invitrogen, Paisley, UK). CD8 labelling was performed using rabbit IgG anti-CD8 (Abcam) at a concentration of 1 : 100 and was detected using goat anti rabbit-IgG Alexa Fluor 488 at 3 *μ*l per 1000 *μ*l. Nuclei were stained with DAPI. Five microscopic fields were imaged in a step-wise manner for each cellular phenotype investigated in the VIN biopsies. In most cases, this covered almost the whole specimen, as the sections were small punch biopsies. The TIL counting was then performed using Image J image analysis software version 1.36b (http://rsb.info.nih.gov/ij/), using the cell counting feature of the software. An independent observer reviewed a range of slides and the counts differed by no more than 5%. Results were reported as density per unit area of CD4, CD8 and double-stained FoxP3CD4 cells.

### Peripheral immune response

Peripheral immune response to HPV antigens was assessed by lymphoproliferative assays. Peripheral blood mononuclear cells (PBMCs) alone or PBMCs with 25 *μ*g ml^–1^ recombinant HPV antigens – HPV16 E6E7L2 protein TA-CIN (Xenova Research Ltd., Cambridge, UK), HPV16 GST E6, HPV16 GST E7 (Smyth *et al*, 2004), HPV16L2 (full-length HPV16L2 tagged with 6His at N-terminus) and TA-GW (HPV 6 L2E7; Xenova Research Ltd.) – and 2 *μ*g well^–1^ PHA were performed as previously described (Winters *et al*, 2008). Responses were measured by tritiated thymidine incorporation after a 5-day incubation of patient PBMCs with the different antigens. Results are presented as stimulation index (SI), defined as the mean number of counts incorporated by the antigen-stimulated PBMCs divided by the mean number of counts for PBMCs in medium alone. A pre-existing proliferative T-cell response was defined as the SI of ⩾2. A post-vaccination proliferative T-cell response was defined as a two-fold increase in the SI compared with the pre-vaccination value.

Neutralising antibodies to HPV16 pseudovirions were tested as by the SEAP pseudovirus neutralisation-based assay as previously described (Karanam *et al*, 2009).

### Statistical analysis

Analyses were performed using ‘Stats Direct Statistical Software’ (StatsDirect Ltd, Altrincham, UK). Differences between responder and non-responder categories (baseline disease duration; HPV16 positivity; smoking; symptom response at week 52 compared with baseline) were analysed using Fisher's exact test. The non-parametric Mann–Whitney *U*-test was used to test for differences in proliferative and lesion T-cell density between groups (lesion responders and non-responders). The non-parametric Wilcoxon's signed-ranks test was used to analyse differences in the responses within groups at different time points. All reported *P*-values are two sided and have not been adjusted for multiple comparisons. A *P*-value of ⩽0.05 was considered to indicate statistical significance.

## Results

A total of 20 women consented to the trial. One woman, who was found to have early stromal invasion on the first study biopsy, was withdrawn from the study and excluded from the analysis, which left 19 women. Patient demographics are presented in Table 1. The group had a mean age of 46 years with average disease duration of 7 years (range 1–20 years). Thirteen patients had been previously treated and seven had at least three previous treatments. Only 4 out of 19 (21%) never previously smoked cigarettes.

### Tolerability

The majority of women experienced local inflammation, ulceration, malaise and flu-like symptoms within the initial few weeks of imiquimod. Side effects were classed as severe in 14 and moderate in 5 women. To improve tolerance, treatment-free intervals of no more than a few days were allowed to achieve the total dose, but to let the side effects subside. Out of 19 women, 16 (84%) completed the prescribed course of imiquimod treatment. Three women noted a marked improvement in facial acne with fewer spots and smoother skin in general while on imiquimod treatment. TA-CIN, which was administered intramuscularly into the deltoid muscle, was well tolerated with no side effects or adverse events.

### Response rates

**Figure 1:**
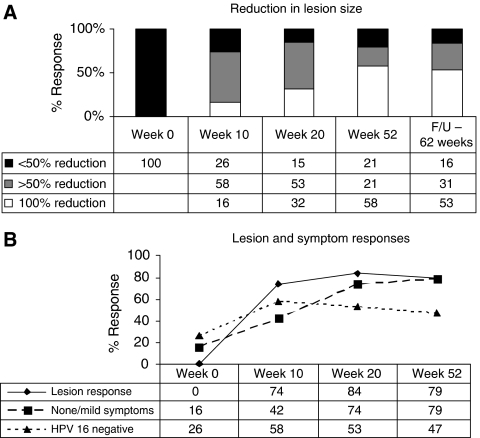
(**A**) Reduction in lesion size. Reduction in lesion size by 100%, >50% but <100 and <50% at baseline (week 0), after imiquimod (week 10), after vaccination (week 20), primary end point (week 52) and follow-up (62 weeks after primary end point) in the group (*n*=19). (**B**) Lesion and symptom responses. Lesion response (complete disappearance of VIN on histology), symptom response (regression to mild/none symptoms), absence of HPV16 on biopsy at baseline (week 0), after imiquimod (week 10), after vaccination (week 20) and primary end point (week 52) in the group (*n*=19).

Table 2 reports the VIN lesion size, histology, HPV status and any patient symptoms at baseline (week 0), after imiquimod (week 10), after vaccination (week 20) and at 52 weeks. Figure 1A stratifies lesion size reduction at weeks 10, 20 and 52 compared with week 0. For example, 74, 85 and 79% of women had a ⩾50% reduction in the size of lesion at weeks 10, 20 and 52, respectively. However, patient 8 still had a visible VIN-like lesion at week 52 but the comprehensive histological analysis indicated resolution of VIN (Table 2). A background of inflammation and pigmentation can confound the true assessment of any lesion and emphasises the need for histological definition of VIN. Figure 1B summarizes the lesion response and HPV status as well as patient symptom responses with time. Complete regression of VIN on histology was 32, 58 and 63% at weeks, 10, 20 and 52, respectively. At baseline, 15 out of 19 (79%) women had moderate-to-severe symptoms compared with 11 out of 19 (58%) at week 10; there was significant reduction at week 20 to 5 out of 19 (26%) and at week 52 to 4 out of 19 (21%), (*P*=0.01). At 0, 10, 20 and 52 weeks, 14, 8, 9 and 10 of the 19 women were HPV16-positive, respectively, and 16 out of 19 were HPV16 positive on at least one occasion. Three women were consistently HPV16 negative but two showed other HPV-type infection at some point. Of the women who showed lesion responses at week 52, 5 out of 10 also cleared their HPV16 infection compared with only 1 out of 6 non-responders; however, this did not reach statistical significance. There were no statistical differences in the lesion responder and non-responder groups with respect to disease duration (Table 1) at trial entry (*P*=0.58) or HPV16 detection at baseline (*P*=0.66). Out of 19 women, 12 were active smokers during the trial period. Current smoking habit was not associated with lesion response (*P*=0.1).

Extended follow-up of these patients for an average of 15 months beyond the primary end point of week 52 showed 84% of patients with a ⩾50% reduction in lesion size consistent with continuing control of VIN (Figure 1A). Unfortunately, biopsy of any index lesion after week 52 was not performed unless clinically indicated. Overall, one patient developed microinvasive disease (patient 7), two had new lesions after week 52 (patients 8 and 17) and one had a recurrence 6 months after the trial end point (patient 14) (Table 2).

### Systemic immune response to HPV16

Pre-existing HPV16 antibodies correlated with documented HPV16 exposure in 12 out of 15 cases. Overall patient pre-existing neutralising antibody levels did not differ or alter significantly after imiquimod or vaccination in either the lesion responders who cleared VIN histologically or the non-responders who failed to clear VIN (data not shown).

**Figure 2:**
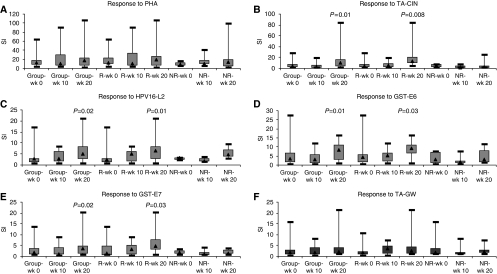
Box-feather plots showing median and quartile lymphoproliferation responses. The figures show stimulation indices of proliferation in response to PHA (**A**), TA-CIN (**B**), HPV-16L2 (**C**), GST-E6 (**D**), GST-E7 (**E**) and TA-GW (**F**) in the whole group (*n*=19), lesion responders (R; *n*=12) and lesion non-responders (NR; *n*=7) at baseline (week 0), after imiquimod (week 10) and after vaccination (week 20). *P*-values that are statistically significant for stimulation indices (SI) at week 20 compared with week 0 are shown. The Wilcoxon's signed-ranks test was used to determine the significance of within-group differences before and after treatment. A *P*-value of ⩽0.05 was considered significant.

Lymphoproliferation to HPV antigens was used to analyse patient systemic cellular immunity to HPV before and after the imiquimod and vaccination steps in the protocol. In all, 16 women (84%) showed pre-existing lymphoproliferative response to TA-CIN (SI of ⩾2), of whom 14 were HPV16 positive on vulval biopsy on at least one occasion. Of these, 11 were lesion responders and 5 non-responders with no significant difference in magnitude of pre-existing response (*P*=0.4). After vaccination, 10 out of 12 lesion responders compared with 3 out of 7 non-responders showed more than two-fold SI change compared with baseline. Figure 2 shows box-plots of the median and quartile lymphoproliferative responses of all the patients as well as stratification into lesion responders or non-responders to the different antigens, TA-CIN vaccine (HPV16 E6E7L2 fusion protein), its component HPV16 antigens L2, E6 and E7 as GST fusion proteins, TA-GW (HPV6 L2E7 fusion protein) as a negative control and PHA as a positive control for lymphocyte proliferation. Strong PHA responses were showed by all the women with median SIs of 12, 10.6, 16.9 at week 0, 10 and 20, respectively; however, these responses were not significantly different after imiquimod or vaccination (*P*=0.4 and *P*=0.2 in the group) or when patients were stratified by lesion response (*P*=0.7 and *P*=0.5 in responders and *P*=0.3 and *P*=0.3 in non-responders) (Figure 2A). In contrast, a significant increase in proliferation to TA-CIN was observed in patients after vaccination compared with pre-treatment (*P*=0.01) and this was associated with lesion responders (*P*=0.008) but not the non-responders (*P*=0.7) (Figure 2B). Similar significant patient proliferative responses to each of the individual HPV16L2, E6 and E7 antigens after vaccination were observed (*P*=0.02, *P*=0.01 and *P*=0.02, respectively), with this increased proliferation associated with lesion responders (*P*=0.01, *P*=0.03 and *P*=0.03) and not non-responders (*P*=0.5, *P*=0.5 and *P*=0.2) (Figures 2C–E). Specificity of the HPV16 responses was supported by the absence of any significant increase in patient lymphoproliferation after vaccination to TA-GW (HPV6 L2E7), in the responder (*P*=0.4) or the non-responder (*P*=0.9) groups (Figure 2F). No significant increase in proliferation to any of the antigens was noted after imiquimod in any of the patient groups. Post-vaccination proliferative response to TA-CIN and its components seems to correlate with the clearance of VIN on histology.

### Tumour-infiltrating lymphocytes

**Figure 3:**
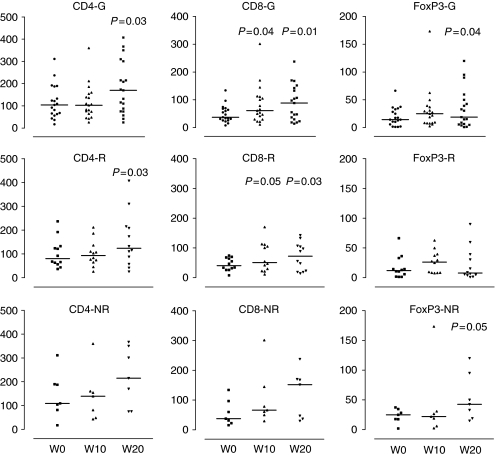
Median/scatter-dot plot of lesion-associated immune cells in the group (G), lesion responders (R) and non-responders (NR). The figure shows median number (density per unit area) of CD4, CD8 and FoxP3CD4 cells before treatment (week 0), after imiquimod (week 10) and after vaccination (week 20) in the group (G), lesion responders (R) and non-responders (NR). The *P*-values for statistically significant difference in the number of cells either at week 10 or week 20 compared with week 0 are shown. The Wilcoxon's signed-ranks test was used to determine the significance of within-group differences before and after treatment. A *P*-value of ⩽0.05 was considered significant.

To analyse local immune factors in the vulval biopsies before treatment, and after imiquimod and vaccination, the densities per unit area of CD4, CD8 and double-stained FoxP3CD4 (T regulatory) cells were assessed. Imiquimod treatment was expected to enhance the local immune infiltration. The data are presented in Figure 3 as median/scatter plots for all the patients as a group or stratified by lesion response. In the group as a whole, significant increases in the number of CD4, CD8 and FoxP3CD4 T cells were evident by week 20 compared with baseline (*P*=0.03, *P*=0.01 and *P*=0.04, respectively); a significant increase in CD8 density was apparent after imiquimod (*P*=0.04). At week 20, the increased CD4 and CD8 density was significantly associated only with the lesion responders (*P*=0.03 and *P*=0.03) whereas increased FoxP3CD4 density was associated with the patients who did not show lesion response (*P*=0.05). There were no significant pre-treatment differences in the density of CD4 or CD8 T cells in lesion responder and non-responders (*P*=0.2 and *P*=0.5, respectively) but intralesional FoxP3CD4 density was significantly higher in non-responders compared with responders (*P*=0.05). By week 20, a significant reduction in FoxP3CD4 T-cell population density was apparent in lesion responders, which was significantly different from non-responder densities (*P*=0.01).

## Discussion

This study was designed on the premise that imiquimod and therapeutic HPV vaccination could combine to alter the balance of local immunity through inducing a local inflammatory environment and enhancing T-cell responses to HPV E6 and E7 proteins. Immunotherapies that tip the balance of immune equilibrium in favour of the host effector response and away from regulatory and viral evasion strategies of the HPV may be the key to enhancing the cell-mediated immunity required to eradicate persistent HPV infection and established disease. This study showed that imiquimod followed by vaccination achieved histological clearance of VIN at 52 weeks in almost 60% of a heavily pre-treated cohort of women with high-grade, long-standing VIN.

With the proviso that the study size was small, the analyses of lesion-associated T cells showed that higher pre-existing and post-treatment levels of regulatory T cells are associated with a lack of lesion response to treatment. As expected in the group as a whole, imiquimod treatment induced T-cell infiltration, which was most apparent by week 20. However, the increased CD4 and CD8 T-cell density was significant only in the lesion responder group, whereas a significantly higher regulatory T-cell density was only observed in the non-responder group. These observations are consistent with immune control and therapeutic effect reflecting the balance of useful CD4- and CD8-directed effectors against their control by T regulatory activity. Thus, increasing the CD8 and CD4 T-cell density may be able to re-establish local immune control that is lost or suppressed in chronic VIN (Gul *et al*, 2004). Where the T regulatory cells dominate the local immunological milieu, they can continue to suppress the HPV antigen-specific cytotoxic T-cell response facilitating persistent VIN (Kobayashi *et al*, 2004; Stanley, 2008). T regulatory depletion before imiquimod or therapeutic vaccination, particularly in women with higher proportion of systemic or intralesional T regulatory cells before treatment, might enhance the stimulation and efficacy of the useful HPV-specific effector T cells. Indeed, there have been several attempts to improve the outcome of different vaccination regimes either by downregulating or blocking T regulatory cells with anti-CD25 antibodies (Chuang *et al*, 2009) or IL-2 diphtheria toxin conjugates (Dannull *et al*, 2005).

The TA-CIN vaccination component of the protocol was aimed at expanding E6- and E7-specific T effectors with the possibility that after imiquimod their entry and activity in the VIN lesions would not be limited by local immunosuppressive factors. Lymphoproliferation of PBMCs established that all patients were immunocompetent and that the vaccination was immunogenic and HPV16 antigen specific. Importantly, these systemic immune responses to HPV16 antigens were significantly associated with lesion responders and not the non-responders. There was no influence on immunity to HPV as a result of the imiquimod treatment. However, a trend towards higher pre-existing responses to HPV16 early antigens in the lesion responder group was noted in this study, which was significantly stimulated by the vaccination. In contrast, the lesion non-responders showed little pre-existing response and this was not boosted by the vaccine. Previous studies have noted a significant correlation between pre-existing systemic HPV16-specific T cells and regression of HPV16-positive lesions (Van Poelgeest *et al*, 2005; Winters *et al*, 2008). The immune response to the HPV vaccine used in this study might have additional effect if delivered with an adjuvant that can boost both serological and cellular immune responses to its HPV16 antigens (Karanam *et al*, 2009).

Overall, it seems that the natural history of the VIN in the non-responders is related to modulation of both local and systemic immune responses allowing persistence of HPV infection. The mechanisms underlying this chronic VIN state are not known but might include genetic predisposition involving immune and other parameters (Davidson *et al*, 2003b). In chronic VIN the suboptimal stimulation of HPV immunity probably also leads to anergy of HPV-specific T effectors. The precise time course of immune responses relevant to HPV lesion clearance are completely unknown and there is no *a priori* reason why it would achieve its full potential at the arbitrary study end point especially for chronic lesions, which have been present for many years. Thus, although some of the histological and symptom response noted at week 52 was evident from week 10 (after imiquimod), the response enhanced with time. It is reasonable to argue that an immunologically challenged chronic condition that takes time to establish will equally take time to regress, explaining the continuing response noted long after treatment completion. In this study of a heavily pre-treated cohort of women with long-standing disease, containment of response for 2 years is noteworthy. Patient 5 (40 years, VIN for 20 years and 7 surgical excisions) and patient 19 (35 years, multifocal VIN for 10 years and 10 surgical treatments) illustrate particularly effective lesion responses achieved by week 52 in this study, and that have been maintained for a further 2 years thus far. In both these cases, intralesional pre-treatment T regulatory cell density was low, with no enhancement after treatment, contrary to increased density of CD4 and CD8 cells. A pre-existing proliferative response to HPV16 with significant increase in proliferation response after vaccination was also showed. It would be beneficial if the likelihood of response to treatment could be determined using biomarkers either before treatment or, in the case of combined therapy, after imiquimod to avoid subjecting likely non-responders to unnecessary treatment. At face value, pre-treatment assessment of dominant immunological cell types in the lesion microenvironment and analysing pre-existing immunity to HPV could provide the basis for selecting patients most likely to benefit from imiquimod with or without vaccination regimes.

In considering the imiquimod treatment, tolerability is a significant issue as the majority of women experience local and systemic side effects lasting for the duration of imiquimod treatment, which may affect daily activities. Overall, our regimen was feasible but, as expected, was associated with considerable imiquimod-induced discomfort necessitating breaks in treatment. Women with refractory VIN are, however, highly motivated to comply with the treatment protocol, and in the event, 85% persevered with treatment and finished the full course of imiquimod. In this study, treatment by 8 weeks of imiquimod followed by vaccination gave a lesion response rate of 63% at week 52. This compares very favourably with imiquimod alone treatment of 16 weeks assessed as histological regression to VIN1 or better as 64% soon after treatment (Le *et al*, 2007) or with no VIN at 2 months after treatment as 81% (Mathiesen *et al*, 2007) or 69% (van Seters *et al*, 2008).

A recent study of VIN patients treated by 3 or 4 immunizations at 3 weekly intervals with a HPV vaccine composed of long HPV16 E6/E7 peptides in adjuvant (Montanide ISA-51, Seppic) showed 60 and 79% lesion size response rate (⩾50% reduction) at 3 and 12 months of follow-up (Kenter *et al*, 2009). As suggested by Kenter *et al* (2009) imiquimod might be more beneficial if used after therapeutic HPV vaccination, as studies indicate that imiquimod treatment may depend on IFN-*γ* producing HPV-specific T cells.

Following another recently published study of imiquimod followed by PDT (Winters *et al*, 2008), continued surveillance for up to 3 years (S Daayana, unpublished results) has shown a sustained clinical response rate of 65% at follow-up. This treatment is now being offered to women in our unit for whom surgical therapy is not suitable. Distinguishing the contribution of the individual components of such combination regimes and establishing proof of either additive or synergistic effects will require further innovative trials. All the recent studies of immunologically driven treatments of VIN provide momentum for further multicentre randomised trials with consistency in measurement of outcomes and definitions of response. Comparing upfront surgical treatment with imiquimod or other potentially more potent TLR agonists (Fahey *et al*, 2009) or therapeutic HPV vaccination (Karanam *et al*, 2009; Kenter *et al*, 2009), or a combination of TLR agonists with therapeutic HPV vaccination in a crossover study design will be valuable.

## Figures and Tables

**Table 1:** Patient demographics

**Patient no.**	**Age (years)**	**Focality**	**h/o CIN**	**Smoking**	**Prev treatment**	**Disease duration (years)**
1	51	M	Yes	No	LASER Vulvectomy Imiquimod	4
2	43	M	Yes	Yes	LASER × 4	9
3	45	M	Yes	Ex	Excision	3
4	40	M	Yes	Yes	Excision	10
5	40	M	No	Yes	Excision × 7	20
6	65	M	No	Ex	None	4
7	58	M	Yes	Yes	Excision × 3 Vulvectomy	15
8	27	M	Yes	Yes	None	1
9	51	M	Hyst	Ex	Diathermy	1
10	55	U	Yes	Yes	Excision	6
11	51	U	No	Yes	LASER × 2	16
12	47	U	Yes	No	None	1
13	56	M	Yes	Yes	None	2
14	43	M	Hyst	No	LASER × 2 Excision × 1	4
15	48	U	No	No	None	1
16	34	M	No	Yes	LASER Imiquimod	3
17	22	M	No	Yes	None	1
18	66	M	Hyst	Yes	Excision × 5 LASER × 4	16
19	35	M	Yes	Yes	Excisions × 6 LASER × 4	9

Abbreviations: CIN=cervical intraepithelial neoplasia; Ex=ex-smoker; Hyst=h/o hysterectomy; Prev=previous; M=multifocal; U=unifocal.

**Table 2:** Clinical responses in VIN patients treated by imiquimod and TA-CIN

	**Lesion size in mm**				**Histology**				**HPV type**				**Symptoms**			
**Patient no.**	**Wk 0**	**Wk 10**	**Wk 20**	**Wk 52**	**Wk 0**	**Wk 10**	**Wk 20**	**Wk 52**	**Wk 0**	**Wk 10**	**Wk 20**	**Wk 52**	**Wk 0**	**Wk 10**	**Wk 20**	**Wk 52**
1*δ*	80	35	30	40	VIN1/2/3	VIN1/2/3	VIN2/3	VIN2/3	Neg	Neg	16	16	Mild	Moderate	Mild	Moderate
2*δ*	75	60	70	70	VIN3	VIN1/2/3	VIN2/3	VIN2/3	16	16	16	16	Severe	Moderate	Moderate	Severe
3	90	65	40	30	VIN2/3	VIN2/3	VIN2/3	VIN1/2	42	Neg	Neg	Neg	Moderate	Severe	Mild	Mild
4*δ*	50	40	30	45	VIN2/3	VIN2/3	VIN2/3	VIN2/3	16	16	16	Neg	Severe	Moderate	Mild	Mild
9	>100	25	20	10	VIN3	VIN2/3	VIN2/3	VIN2/3	16	16	16	16	Severe	Moderate	Mild	Mild
16*ϕ*	45	20	30	40	VIN2/3	VIN2/3	VIN2/3	VIN2/3	16	16	16	16	Severe	Severe	Severe	Severe
18	30	20	10	20	VIN2/3	VIN2/3	VIN2/3	VIN2/3	16	16	16	16	Mild	Mild	Mild	Mild
5	60	10	0	0	VIN1/2/3	No VIN	No VIN	No VIN	16	Neg	Neg	Neg	Mild	Moderate	None	None
6	65	25	5	0	VIN2/3	No VIN	No VIN	No VIN	16	16	Neg	16,53	Severe	Mild	None	None
7*χ*	100	50	50	0	VIN3	No VIN	No VIN	No VIN	16,33,84	Neg	N/E	33	Severe	Mild	Mild	Mild
8*α*	60	25	25	25	VIN2/3	VIN2/3	VIN2	No VIN	16	16,33,81	N/E	16	Severe	Severe	Severe	Severe
10*δ*	25	10	0	0	VIN2/3	VIN1/2/3	No VIN	No VIN	Neg	Neg	Neg	Neg	Severe	None	None	Mild
11	25	0	0	0	VIN3	No VIN	No VIN	No VIN	Neg	Neg	84	84	Moderate	Moderate	None	None
12	20	0	0	0	VIN3	VIN1/2	No VIN	No VIN	Neg	33	Neg	16,33	Moderate	Moderate	None	None
13	50	40	20	0	VIN3	VIN1/2	No VIN	No VIN	16	16	16	16	None	Mild	None	None
14*β*	90	15	15	0	VIN2/3	VIN1/2	No VIN	No VIN	16	Neg	Neg	Neg	Moderate	None	Moderate	Mild
15	25	3	0	0	VIN3	No VIN	No VIN	No VIN	16	Neg	16	Neg	Severe	None	None	None
17*α*	40	3	4	0	VIN2	VIN1/2	No VIN	No VIN	16	Neg	16	16	Moderate	Moderate	Moderate	Mild
19	>100	0	0	0	VIN2/3	No VIN	No VIN	No VIN	16	Neg	6	6	Severe	Mild	Mild	None

Abbreviations: HPV=human papillomavirus; CIN=cervical intraepithelial neoplasia; Neg=negative; N/E=non-evaluable; VIN2/3=vulval intraepithelial neoplasia grades 2 and 3; wk=week.

Patient 7 developed ESI (*χ*) at week 30; patients 1, 2, 4 and 10 underwent LASER treatment (*δ*) after trial completion; patient 14 developed recurrence (*β*) 6 months after trial completion and underwent LASER and surgery. Patients 8 and 17 developed new lesions (*α*) after week 52 treated with LASER. Patient 16 repeated course of imiquimod (*ϕ*) after trial completion.
